# Current Perspectives in Detection of Bioactive Compounds From *Costus spicatus* Through GC–MS and LC-MS/MS: Antioxidant Properties and In Silico Analysis for Industrial Applications

**DOI:** 10.1155/bri/9599942

**Published:** 2025-01-15

**Authors:** Dharmar Manimaran, Ararsa Tessema, M. Govindarajulu Yadav, S. Parthasarathi, Vasan Palanisamy

**Affiliations:** ^1^Department of Animal Nutrition, Veterinary College and Research Institute, Tamil Nadu Veterinary and Animal Sciences University, Chennai, Tamil Nadu, India; ^2^Department of Food Engineering, Arba Minch University, Arba Minch, Ethiopia; ^3^Department of Biotechnology, Paavai Engineering College, Namakkal, Tamil Nadu, India

**Keywords:** antioxidant activities, bioactive compounds, *Costus spicatus*, GC–MS, LC-MS/MS

## Abstract

Members of the *Costus* genus are the conventional medicinal plants used in the therapeutic management of numerous ailments, especially for their antioxidant and pharmacological activities. The crude extract of *Costus spicatus* was profiled using high-resolution GC–MS and LC-MS/MS techniques to determine possible bioactive compounds that are vital to the antioxidant activity. A total of 52 and 63 bioactive compounds have been detected in GC–MS chromatograms using different solvents (methanol and ethanol) in *C. spicatus* leaf extracts, representing the presence of certain bioactive compounds. The identified bioactive compounds in both extracts which exhibit neuroprotective effects have been confirmed through various literature studies. They are cholestan-3-amine, loliolide, stigmasterol and methylprednisolone acetate, succinimide, fumaric acid, beta-tocopherol and gamma-tocopherol. The aqueous extract possessed the highest antioxidant activity for DPPH scavenging activity and lipid peroxidation inhibition assays, whereas the alcoholic aqueous extract showed superior efficacy for hydroxyl radical scavenging activity. In this study, we performed molecular docking and found that four compounds exhibited promising binding affinities with predicted binding sites on alpha-synuclein. Notably, Androsta [17-16-b] furan-5′-imine, 4′-methylene-3-methoxy-N-cyclohexyl- showed the highest docking interaction score of −7.4, indicating a strong binding affinity. These findings, combined with the presence of bioactive components in the crude extract of *C. spicatus*, suggest that this plant may possess neuroprotective properties, warranting further investigation for potential industrial applications in the development of neuroprotective agents.

## 1. Introduction

Progressive loss or even death in the structure and function of neurons is the characteristic feature of neurodegenerative diseases, which has a significant adverse effect on both humans and society. Healthcare professionals are still uncertain of the precise causation of certain neurodegenerative illnesses. Protein destruction, oxidative stress, inflammation, lifestyle exposures, mitochondrial defects, genetic factors and aberrant protein build-up in neurons are a few of the often-investigated environmental root causes of neurodegenerative illnesses [1]. Natural supplements are actively employed as a kind of holistic therapy due to their multicomponent strategy to target many areas for their mechanism of action [2]. Furthermore, their composition in a single delivery mode and lack of adverse effects make them potential candidates for treating a variety of central nervous system (CNS) disorders [3]. These herbal preparations can be consumed alongside other medications without inducing any adverse interactions between drugs [4].

Herbal products include complex blends of organic substances such as alkaloids, flavonoids, fatty acids, sterols and so on. In comparison with the isolated compounds found in conventional medications identified by pharmacologists, advocates of herbal remedies assert that a plant's healing properties result from the collaborative impact of its various components. As a result, there is a belief that traditional medicines are effective and have minimal to no adverse effects [5]. Pharmaceuticals and biotech industries widely use natural plant products as many modern medicines are derived from their molecules or derivatives [6]. Medicinal food plants offer curative benefits, leading to the term functional food, which modulates specific body functions [7]. In recent years, plant secondary metabolites have attracted attention for their antioxidant, anti-inflammatory and anticancer properties [8]. Plant-derived bioactive compounds are valuable for drug, nutraceutical and cosmeceutical development, but selecting the appropriate plant part and extraction method is crucial [9]. The therapeutic efficacy of medicinal plants is often correlated with their reservoir of diverse secondary metabolites. Phenolic acids in plants are considered one of the most promising groups of secondary metabolites owing to their plethora of biological and pharmacological attributes mostly linked with their antioxidant properties [10]. Certain species are known for their rich content of secondary metabolites, including volatile oils, sesquiterpene lactones, diterpenes, triterpenes, lignans, flavonoids, phenolic acids as well as amino acids, fatty acids, alkanes and inulin [11].

Identifying and characterizing novel medicinal plants to treat neurodegenerative ailments and brain damage caused by stroke has been a significant and expanding recent scientific area of focus. In Asian nations, over 120 traditional medicines are utilized for the treatment of CNS diseases. Researchers have been trying to find therapeutic interventions for neurodegenerative ailments using natural chemicals derived from plants more frequently in recent years [12]. Various medicinal plants have demonstrated potential efficacy in neuropsychopharmacology in the Indian System of Medicine [13]. Chia seeds have numerous beneficial antioxidant phenolic components and are used to make a variety of functional meals and beverages. Chia seeds also suppress cholinesterase activity, provide protection against neurological disorders and enhance insulin sensitivity and lipid metabolism [14].

As a result, based on several ethnomedical claims, we have selected the *Costus spicatus* plant, which is prolific in secondary metabolites that are detected in a variety of preparations. Studies in preclinical pharmacology have revealed analgesic, anti-inflammatory, nephroprotective and antilithiatic, antidiabetic and antifungal actions [15]. According to ethnopharmacological studies, *C. spicatus* leaf and stem extracts, as well as aqueous or alcoholic infusions, are frequently used to treat cutaneous ulcers, renal calculi, infections, inflammation, gonorrhoea, urethritis and leucorrhoea [16]. These benefits have motivated researchers from a variety of fields, including chemistry and pharmacology, to thoroughly screen plants as potential replacements for synthetic pharmaceuticals already active [17].

Medicinal and aromatic plants are rich in diverse phytochemicals, requiring thorough screening to find compounds beneficial for medicine, cosmetics and food industries. Generally, only a small number of these compounds show bioactivity and are used industrially [18]. Studies use liquid chromatography electrospray ionization tandem mass spectrometry for quantitative analysis, developing a method for different compounds [19]. LC-MS/MS identified quinic, malic and chlorogenic acids, rutin and hesperidin as major phenolics in extracts, with methanol being most effective [20]. Utilizing several experimental animal methods, ethnopharmacological work has been refined continuously to look for novel and powerful therapeutic compounds from plant sources for the treatment of several ailments, particularly neurological diseases. Therefore, increased attention has been given to standardizing treatments for these ailments using *C. spicatus* plants. Despite the extensive research, there are no reported studies on the neuroprotective activity of *C*. *spicatus*. In general, several solvents were employed to extract plant metabolites from their various components. Furthermore, the extraction of phytocompounds and yield are heavily influenced by the kind of solvents used and the extraction process [21]. The existing literature reveals that no studies have been conducted to compare the effects of different solvents on the biochemical composition of *C. spicatus* plant extracts.

*In silico* virtual screening has become a reliable, cost-effective and timesaving technique that is complementary to in vitro screening for the discovery and optimization of potent lead and hit compounds. Nowadays, these approaches have paved the way to solve many biological issues, which have led to the identification of novel inhibitors against numerous diseases [22]. Therefore, the present study focuses on the detection of bioactive compounds from C. *spicatus* using GC–MS and LC-MS/MS as well as the evaluation of its antioxidant properties and in silico analysis for industrial applications.

## 2. Materials and Methods

### 2.1. Plant Material Collection

**Figure 1:**
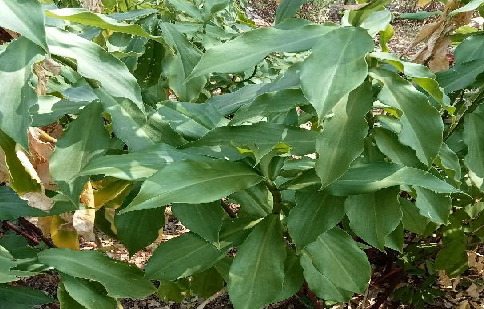
*Costus spicatus* plant.

The leaves of *C. spicatus* were collected in November 2022. The collected plant materials (Figure 1) were identified and authenticated by the Botanical Survey of India (Ref. No. BSI/SRC/5/23/2022/Tech/571). After identification, the leaves were cleaned with running tap water and distilled water, after which they were air-dried in the shade at room temperature. The dried leaves were ground using a laboratory blender. The powdered samples were kept in a refrigerator for further use.

### 2.2. Preparation of Extracts

The powdered leaves of *C. spicatus* were stored in a beaker with 100 mL of organic solvents (methanol and ethanol) and shaken well. The mixture was then left at room temperature for 48 h and stirred 2–3 times each day. The mixture was filtered, and the resulting filtrate was dried using a rotary evaporator. The resulting extracts were weighed to estimate the yield (%), and the dried extracts were kept in a refrigerator at 4°C for future research.

### 2.3. Chemical Component Estimation Using GC–MS

The analysis with GC–MS was conducted utilizing the Clarus 500 PerkinElmer gas chromatograph, which was coupled to a mass detector Turbomas 5.2 spectrometer. The system was equipped with an Elite-1 capillary column (100% dimethylpolysiloxane) measuring 300 m × 0.25 mm × 1 m df. The instrument was configured with an initial temperature of 110°C and maintained at this level for a duration of 2 min. Upon completion of this duration, the oven temperature was increased to 280°C at a rate of 2°C/min and held steady for 9 min. The injection port temperature was established at 250°C, and the helium flow rate was set at 1 mL/min. The ionization voltage was maintained at 70 eV. The samples were introduced in a 10:1 split mode. The mass spectral scanning range was configured from 45 to 600 (m/z). GC–MS was employed for the identification of chemical constituents using the mass spectra fragmentation patterns, which were compared to those archived in the National Institute of Standards and Technology Mass Spectral database (NIST-MS) within the spectrometer database. The percentage of each component was determined by analysing the relative peak area in the chromatogram.

### 2.4. Chemical Component Estimation Using LC-MS/MS Analysis

The injection volume was 5 µL using the autosampler mode and the mobile phase was with 0.1% acetic acid in water (A) and 0.1% acetic acid in acetonitrile (B) in a gradient manner shown in Table 1. The flow rate was 0.7 mL/min. The ambient temperature gradient was as follows: column temperature was 40°C, interface temperature was 300°C, desolvation temperature was 526°C, and heat block temperature was 400°C with a drying gas flow of 10 L/min. Both positive and negative polarities were included in the analysis with the scan module. The generated data were integrated using Shimadzu Lab Solutions software version 6.89.

### 2.5. Antioxidant Activities

#### 2.5.1. Antioxidant Determination via 2, 2-Diphenyl-1-picrylhydrazyl (DPPH) Radical Scavenging Activity

The potential of the extracts to identify and eliminate free radicals was assessed using the DPPH radical scavenging test developed by Blois [23] and Desmarchelier et al. [24]. The ability of plant solvents to contribute hydrogen atoms was assessed by decolorizing a methanol solution containing DPPH. When placed in a methanol solution, DPPH displays a violet/purple colour, which transitions to various shades of yellow when exposed to antioxidants. A 0.1 mM DPPH solution in methanol was made, and 2.4 mL of this solution was combined with 1.6 mL of extract in methanol, ethanol, aquas ethanol and aquas at various concentrations (12.5–150 g/mL). The reaction solution was completely vortexed and placed in the dark at room temperature for 30 min. The absorbance of the solution was measured using a UV–Vis spectrophotometer at 517 nm (Labman, India). BHT was considered a model reference in this study.

(1) $$\begin{array}{c} \text{DPPH radical scavenging activity}=\frac{A_{0}-A_{1}}{A_{0}}, \end{array}$$

where *A*_0_ is the absorbance of the control and *A*_1_ is the absorbance of the extractives/standard.

#### 2.5.2. Hydroxyl Radical Scavenging Activity

The extractives' capacity to scavenge hydroxyl radicals was estimated using the method adopted by Halliwell et al. [25]. The Fe^3+^/ascorbate/EDTA/H_2_O_2_ (Fenton reaction) system resulted in the production of a hydroxyl radical. The methodology of the experiment was based on detecting the 2-deoxy-D-ribose degradation product, which forms a pink chromogen when heated with thiobarbituric acid at a pH below 7. The reaction blend consisted of 0.8 mL of phosphate buffer solution (50 mmol L-1, pH 7.4), 0.2 mL of extractives/standards with diverse concentrations (ranging from 12.5 to 150 g/mL), 0.2 mL of EDTA (1.04 mmol L-1), 0.2 mL of FeCl_3_ (1 mmol L-1) and 0.2 mL of 2-deoxy-D-ribose (28 mmol L-1). The mixtures were placed in a water bath at 37°C, and the reaction commenced with the addition of 0.2 mL of ascorbic acid, AA (2 mmol L-1) and H_2_O_2_ (10 mmol L-1). After 1 h of incubation at 37°C, 1.5 mL of chilled TBA (10 g L-1) was added to the reaction mixture, followed by 1.5 mL of HCl (25%). The material was subjected to a temperature of 100°C for a duration of 15 min and subsequently cooled using water. A spectrophotometer was used to measure the absorbance of the solution at 532 nm.

(2) $$\begin{array}{c} \text{Hydroxyl radical scavenging activity}=\frac{\left[A_{0}-A_{1}-A_{2}\right]}{A_{0}}, \end{array}$$

where *A*_0_ is the absorbance of the control without a sample, *A*_1_ is the absorbance following the addition of the sample and 2-deoxy-D-ribose, and *A*_2_- is the absorbance of the sample without 2-deoxy-D-ribose.

#### 2.5.3. Lipid Peroxidation Inhibition Assay

The assay for lipid peroxidation inhibition was performed using the methodology established by Haenen and Bast [26]. To obtain liposomes, the excised rat liver was blended in buffer and centrifuged. To produce a 10% liver homogenate, the extracted Wistar rodent liver (150 g) was chopped in chilled saline solution with phosphate buffer (pH 7.4) using a glass Teflon homogenizer (50 mm). The homogenate was centrifuged at 12,000 rpm for 15 minutes at 4 °C, and the resulting product was used as a liposome in a lab-based study of lipid peroxidation. The blending and filtering activities were carried out in a very frigid temperature state. Initially, 1 mL of 0.15 mg of KCl and 3 mL of extracts or standard at varied concentrations (12.5–150 g per millilitre) were added to 50 mL of supernatant. Lipid peroxidation commenced with 300 L of a solution containing 0.5 mM FeCl_3_. The reaction was halted after 30 min at 37 degrees Celsius by incorporating 2 mL of frigid TBA–TCA–HCl–BHT mixture.

Then, to prepare a TBA–TCA–HCl solution, 1.68 mg TCA and 41.60 mg TBA were dissolved in 10 mL of 0.125 M hydrochloride. For a 10-mL solution of TBA–TCA–HCl, 1 mL BHT solution (1.5 mg/mL ethanol) was added. The mixture was subjected to heating at 90°C for a duration of 60 min, followed by cooling on ice and centrifugation at 3000 rpm for 5 min. The resulting supernatants were withdrawn, and the magnitude of the pink complex was measured at 532 nm using a spectrophotometer. The amount of TBARS found (TBA-reactive chemicals) has been used to determine the level of lipid peroxidation in the body. In the absence of the extract and with the inclusion of distilled water, a control trial was conducted. The proportion of lipid peroxidation reduction in the samples was calculated using the following formula:

(3) $$\begin{array}{c} \text{Lipid peroxidation }\left(\%\right)=\left[\frac{A_{0}-A_{1}}{A_{0}}\right]\times 100, \end{array}$$

where *A*_0_ is the absorbance of the control and *A*_1_ is the absorbance of the extractives/standard.

### 2.6. Computational Feature

All *in silico* analyses were performed using HP Workstation Z220 with next-generation 22 nm processors, including Intel Xeon processor E3-1200v2 family with 16 GB RAM, 1 TB hard disk, NVIDIA Quadro 2000, Windows 7 Ultimate 64 bit. Acclery's Discovery Studio 3.5 (AD 3.5), CLC Genomic Workbench 5.1 and AutoDock Vina software were used for in silico analysis.

#### 2.6.1. Selection of Target Protein

Alpha-synuclein aggregates perturb dopaminergic transmission and induce presynaptic and postsynaptic dysfunctions. Thus, alpha-synuclein was selected as the potential target protein. The 3D structure of alpha-synuclein was retrieved from the PDB database, which is available on the RCSB website (https://www.rcsb.org/).

### 2.7. Statistical Analysis

The antioxidant properties of each extracts were compared by one-way analysis of variance (ANOVA) and p value was established (https://www.statskingdom.com/).

## 3. Results

### 3.1. GC–MS Profiles

#### 3.1.1. Methanolic Extracts

An effective method for identifying the bioactive substances in the natural product is GC. The GC of methanolic leaf extracts of *C. spicatus* revealed 52 peaks, which indicated the presence of 52 chemical components. After comparing the mass spectra of the constituents with those in the NIST library, the 52 different organic chemical compounds were identified and are listed in Table 2. The compounds that occupied the major percentage in the extract are i-propyl 9-octadecenoate (Z) (5.44%), 13-docosenamide (Z)- (11.31%), gamma-sitosterol (21.72%), stigmasterol (9.25%), methylprednisolone acetate (3.22%), alpha-tocospiro B (3.18%) and 9,19-cyclolanostan-3-olacetate (3 beta) (2.38%).

#### 3.1.2. Ethanolic Extracts

The GC–MS chromatogram of ethanolic leaf extracts of *C. spicatus* revealed 63 peaks, which indicated the presence of 63 chemical components. After comparing the mass spectra of the constituents with those in the NIST library, the 63 different organic chemical compounds were identified and are listed in Table 3. The compounds that occupied the major percentage in the extract are 9-octadecenoic acid (Z)-,3-[(1-oxohexadecyl)oxy]-2-[(1-oxooctadecyl)oxy]propyl ester (23.72%), alpha-tocospiro B (13.41%), delta-tocopherol (8.2%), octadecanoic acid, 3-[(1-oxohexadecyl)oxy]-2-[(1-oxotetradecyl)oxy]propyl ester (12%),13-docosenamide (Z) (6.77%), alpha-tocospiro A (6.39%), fumaric acid, decyl 2-fluorophenyl ester (6.15%), hexadecanoic acid, 2-[(1-oxododecyl)oxy]-1,3-propanediyl ester (4.85%), glycine, N-(cyclobutylcarbonyl)-, hepta-decyl ester (4.75%), octadecanoic acid, 3-[(1-oxohexadecyl)oxy]-2-[(1-oxotetradecyl)oxy]propyl ester (4.42%), glycine, N-(cyclo-butylcarbonyl)-, hexadecyl ester (3.23%), hexadecenoic acidic substance, 2-[(1-oxododecyl)oxy]-1,3-propanediyl ester (2.9%) and beta-tocopherol (2.09%).

### 3.2. Antioxidant Properties

The result of the antioxidant property analysis is given in Table 4. The maximum DPPH scavenging activity (2.24 ± 0.02) was recorded in the aqueous extract and the minimum (1.02 ± 0.02) in the ethanol extract of leaves in *C. spicatus*. The maximum hydroxyl radical scavenging activity (1.98 ± 0.002) was obtained in the alcoholic aqueous extract and the minimum (0.75 ± 0.002) in the methanol extract of leaves in *C. spicatus.* The maximum percent lipid peroxidation inhibition assay (42.84 ± 5) was recorded in the aqueous extract and the minimum (28.81 ± 5) in the alcoholic aqueous extract of leaves in *C. spicatus.*

### 3.3. LC-MS/MS Profiles

**Figure 2:**
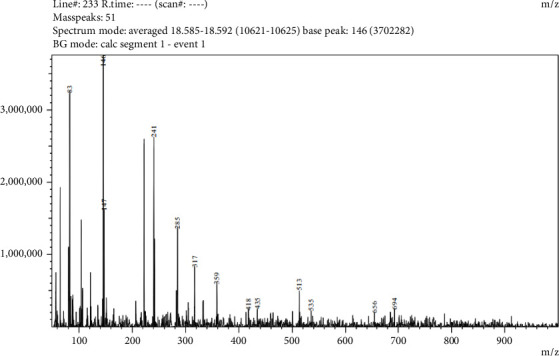
LC-MS/MS zoomed spectrum of caffeoyl shikimate detected from the aqueous extract of *C. spicatus* L. at positive ESI.

**Figure 3:**
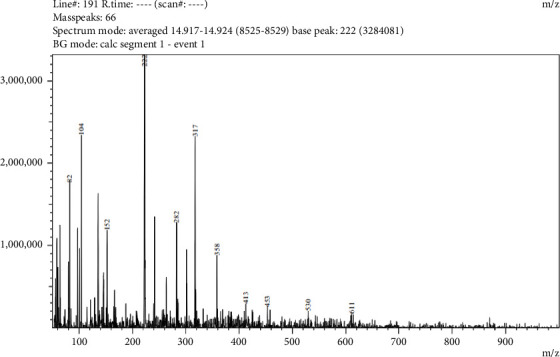
LC-MS/MS zoomed spectrum of *rutin* detected from the aqueous extract of *C. spicatus* L. at positive ESI.

**Figure 4:**
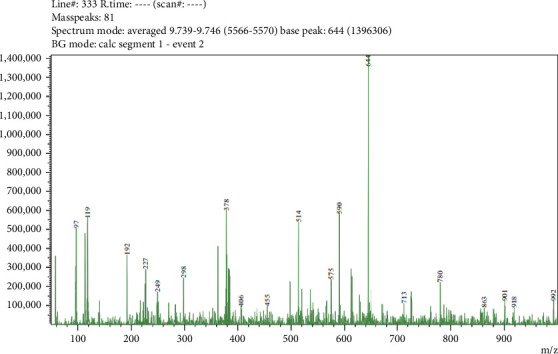
LC-MS/MS zoomed spectrum of *protodeltonin* detected from the aqueous extract of *C. spicatus* L. at positive ESI.

**Figure 5:**
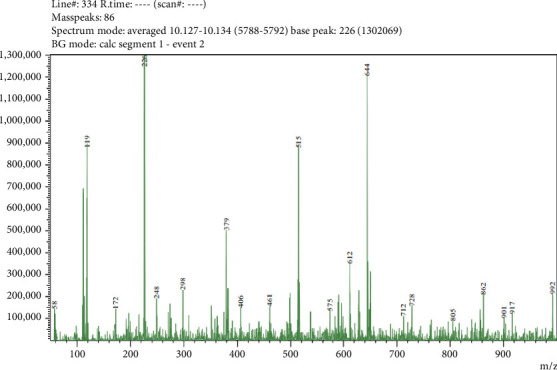
LC-MS/MS zoomed spectrum of *methylprotogracillin* detected from the aqueous extract of *C. spicatus* L. at positive ESI.

**Figure 6:**
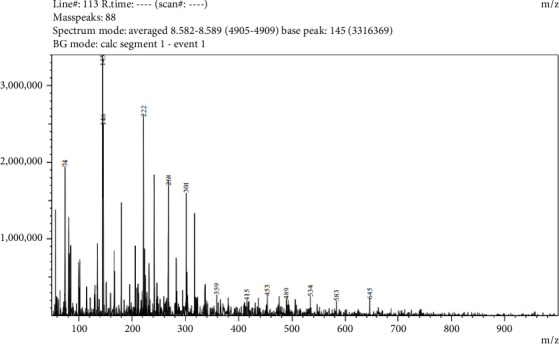
LC-MS/MS zoomed spectrum of *pseudoprotodioscin* detected from the aqueous extract of *C. spicatus* L. at positive ESI.

Five compounds were detected in LC-MS/MS analysis of leaf parts' aqueous extract of *C. spicatus* L. with positive ESI (Tables 1 and 5 and Figures 2, 3, 4, 5 and 6). The compounds identified were phenol, flavonoids, steroids and saponins. By comparing mass spectrum data to the literature [17], all of the chemicals were recognized. The two steroid compounds (protodeltonin and pseudoprotodioscin) were identified with positive ESI (901.6133 and 415.2805) in leaf parts followed by two flavonoid compounds, namely, rutin and methylprotogracillin with positive ESI (611.1602 [M + H]+ and 901.4246) and one phenolic compound (caffeoyl shikimate) with positive ESI (359.0658 [M + Na]+) in leaf parts.

### 3.4. In Silico Evaluation of Compound Interaction on α-Synuclein

**Figure 7:**
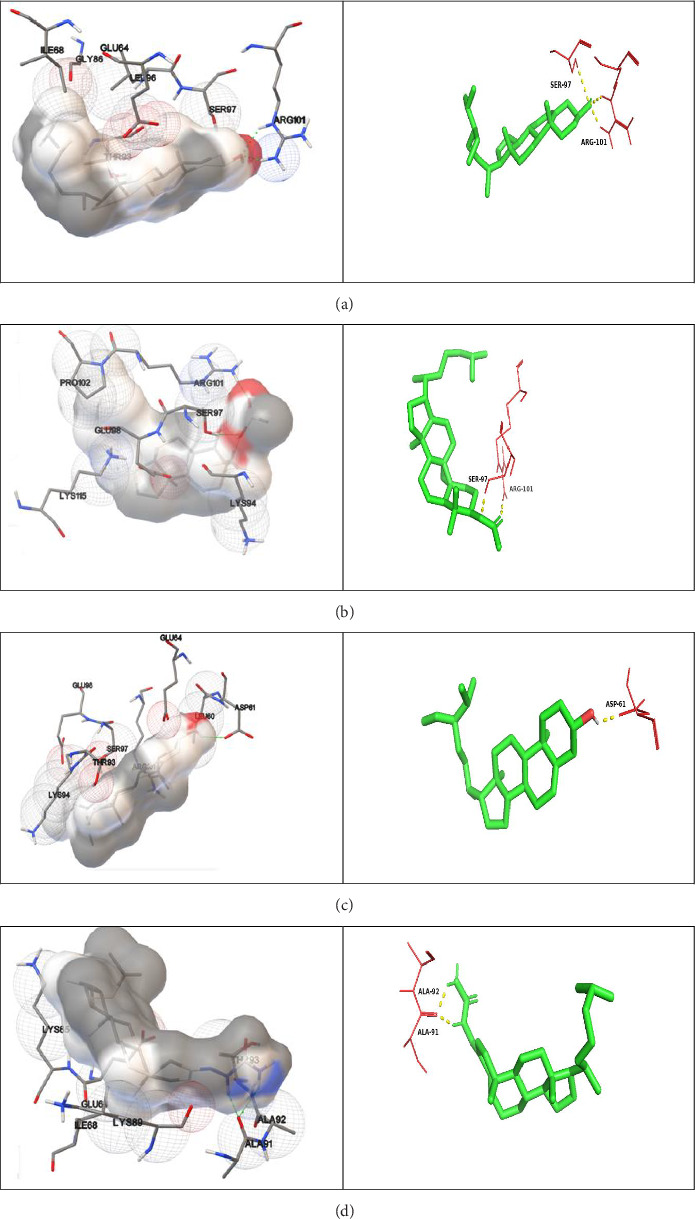
Docking complex and interactions of compound as alpha-synuclein. (a) Androsta [17-16-b] furan-5′-imine, 4′-methylene-3-methoxy-N-cyclohexyl-. Docking complex and interactions of compound with dock score: −7.4 kJ/mol. (b) 9, 19-cyclolanostan-3-ol, acetate (3. beta.). Docking complex and interactions of compound with dock score: −7.2 kJ/mol. (c) i-propyl 9-octadecenoate (Z). Docking complex and interactions of compound with dock score: −6.8 kJ/mol. (d) l-(+)-ascorbic acid 2,6-dihexadecanoate (2d). Docking complex and interactions of compound with dock score: −6.8 kJ/mol.

The extracted compounds were screened through virtual screening methods using AutoDock Vina. The Lipinski's rule of five and drug-likeness properties were also evaluated as shown in Table 6. The screened top five compounds are (i) Androsta [17-16-b]furan-5′-imine, 4′-methylene-3-methoxy-N-cyclohexyl-, (ii) 9,19-cyclolanostan-3-ol, acetate, (3 beta)-, (iii) i-propyl 9-octadecenoate (Z), (iv) l-(+)-ascorbic acid 2, 6-dihexadecanoate (2d) and (v) control (Table 7). Levodopa was docked against the Parkinson disease (PD)-causing receptor (Figure 7).

## 4. Discussion

The several biological activities of the leaf extract are determined to analyse the chemical composition of each extract using GC–MS analysis. Numerous studies have demonstrated the presence of various nonvolatile compounds in different solvents (methanol, ethyl acetate, water and chloroform extracts) derived from diverse medicinal plant parts (leaves, stems and roots) across the world. To the best of our knowledge, there is only a scant report of GC–MS-based metabolite profiling to determine the presence of distinct bioactive chemicals in different solvent extracts of *Costus* plants. The GC–MS study of phytoconstituents in various solvents of *C. spicatus* leaf extracts illustrates the plant's medicinal significance. The bioactive compounds found have a wide range of biological characteristics.

Methanol extracts of the GC–MS profile in the present study with the highest peak area (%) give rise to several compounds (10–12) compared with ethanol extracts. Omer et al. [27] reported that comparing the significant peak area of both extracts (ethanol and aqueous extract) illustrated that ethanol solvent is superior in extracting GC-MS detectable phytoconstituents. A recent study by Zhu et al. [28] reported that palmitic acid suppressed prostate cancer cell proliferation both in vitro and in vivo. In the presence of palmitic acid, inhibiting G1 phase arrest was related to downregulation of cyclin D1 and p-Rb and an increase in p27 expression. In previous studies, palmitic acid has been confirmed to have a wide variety of antibacterial and antifungal properties [29]. Acids such as hexadecenoic acid and 2-hydroxy-1-(hydroxymethyl) ethyl ester were found in both extracts in our study, and these compounds have been regarded as antibacterial agents. Additionally, it has been proven that hexadecenoic acid methyl ester (palmitic acid methyl ester) is a potential neuroprotective molecule in cerebral ischemia [30].

Vitamin E (alpha-tocopherol) is the most important lipid-soluble antioxidant, and it protects cell membranes from oxidation, thus stabilizing them and maintaining their permeability [31]. Vitamin E supplement elevates the activities of antioxidant enzymes [32]. In humans, the high supplementation of vitamin E has been shown to induce pro-oxidant activity, making them react directly with other free radicals or induce lipid oxidation under mild oxidative stress but not under severe situations [33]. In this study, the levels of the vitamin E components, namely, delta- and beta-tocopherol, are significantly higher than gamma-tocopherol in methanolic extracts, but in ethanolic extracts, the concentrations of these compounds are lower.

Phytol is a diterpene molecule that has antibacterial, anti-inflammatory, anticancer and diuretic properties. It also has antiarthritic properties and indicates that reactive oxygen species are a promising new class of medications for the treatment of rheumatoid arthritis and other chronic inflammatory illnesses [34]. Stigmasterol is an unsaturated plant sterol that functions as a precursor in the production of semisynthetic progesterone, a worthy human hormone that has a significant physiological role in the regulatory and tissue-rebuilding mechanisms associated with oestrogen effects. It functions as an intermediate in androgen, oestrogen and corticoid biosynthesis and also is employed as vitamin D3 precursor [35]. GC–MS analysis in our study confirmed that phytol compounds were present in trace amounts in both extracts, whereas stigmasterol compounds were identified only in ethanolic extract with considerable amounts.

Recently, γ-sitosterol, an epimer of β-sitosterol, has been asserted to have an antihyperglycemic effect by enhancing insulin production in response to glucose, as proven by immunological histochemical studies of the pancreas. In this study, γ-sitosterol was found to be the major component with a significant peak area (21.72%) in methanolic extracts. Erucamide, often referred to as 13 docosenamide, is a member of the group of chemical compounds known as fatty amides, which has antibacterial action [36]. This compound was present in both extracts in a respectable proportion with greater amounts in methanolic extracts than in ethanol.

Researchers have been attempting to develop effective treatments for neurodegenerative illnesses using natural substances derived from plants more frequently in recent years. The processes behind the development and progression of PD remain unclear although there is substantial evidence for oxidative stress involved in the destruction of dopaminergic neurons. For neuronal survival, the regulation of the redox potential is crucial, and its disturbance may interfere with other biological functions of the cells, resulting in cell death. Once oxidative stress harms cellular proteins and disturbs lipid membranes, increased ROS, mitochondrial malfunction and neuroinflammation occur in the brain. In order to treat PD, research should be focused on the protective mechanisms that are implicated in the maintenance of these actions. Yet, innovative ideas for this type of study should be guided by the failures of trials with antioxidant compounds and methods performed to date [37].

Based on the above statement, our study identified certain bioactive compounds in both extracts that exhibit neuroprotective effects confirmed through various studies. They are cholestan-3-amine [38], loliolide [39], stigmasterol [40], methylprednisolone acetate [41], succinimide [42], fumaric acid [43], beta-tocopherol and gamma-tocopherol [44].

Due to oxidative stress, free radicals have been associated with a variety of human illnesses including cancer, cardiovascular disease, neurological disorders and others. They can also destroy vital proteins, DNA and lipids. Antioxidants' primary function is to prevent free radical-mediated oxidation by scavenging free radicals before they are produced [45]. Date seeds are high in fibre, polyphenols and antioxidants and are commonly utilized in culinary and medicinal products. Date palm (DP) seed and saw palmetto (SP) seed phenolic-rich extracts reduced diabetes and associated consequences by promoting tissue regeneration and reducing oxidative stress in STZ-induced diabetic rats [46].

In our findings, the maximum antioxidant property for DPPH scavenging activity and lipid peroxidation inhibition assay was obtained in the aqueous extract. In hydroxyl radical scavenging activity, alcoholic aqueous showed higher activity. The contrast pattern of our study with respect to the antioxidant activities of both the extracts (methanol and aqueous) was examined by Semwal and Painuli [47] using two complementary methods, namely, DPPH and hydrogen peroxide (H_2_O_2_) assay. The result showed that the maximum percent DPPH scavenging activity and H_2_O_2_ scavenging activity were recorded in the methanolic extracts. Yao et al. [48] observed that methanol as a solvent resulted in higher values for DPPH and H_2_O_2_ assays, while the aqueous extract resulted in higher values for TFC.

The LC-MS results revealed that aqueous leaf extracts contain various phytocompounds such as flavonoids, steroid saponin and phenols in agreement with previous reports [49]. Flavonoids are found to be higher than all other compounds responsible for elevated response to various infections and damage. Flavonoids are utilized in folk medicines for gastrointestinal, vascular and respiratory diseases. Recent studies have shown that diverse biological activities, such as antioxidant, antidiabetic and anti-inflammatory activities, are associated with the phenolic compound contents found in fruits and vegetables [50]. Therefore, the presence of these compounds in *C. spicatus* may encourage the use of this plant in research and consumption while also providing a basis to support future studies and applications.

Computational techniques such as molecular docking have emerged as robust tools for identifying potent inhibitors and elucidating ligand–receptor interactions in drug design. These approaches complement in vitro and in vivo studies, providing valuable insights into the molecular mechanisms of drug–receptor interactions [51, 52]. By leveraging these computational methods, researchers can accelerate the discovery and development of novel pharmaceuticals, streamlining the drug design process. In this study, it was confirmed that the compound Androsta [17-16-b]furan-5′-imine, 4′-methylene-3-methoxy-N-cyclohexyl- binds specifically to the active site of the alpha-synuclein protein, thereby inhibiting its self-association. This binding activity suggests that the compound has potential as a drug candidate for the treatment of PD.

## 5. Conclusions

The current study finally showed that the solvent used for extraction has a profound impact on the phytochemical profile of *C. spicatus* leaf extracts. Variations in their phytochemical characters demonstrated significant antioxidant activity. With excellent binding potential energy pharmacokinetic/pharmacodynamics modelling properties, virtual hits of these molecules can be considered for early drug development against PD after being tested through in vitro and in vivo studies. Therefore, our research indicates that the leaf extract of *C. spicatus*, which has a high concentration of bioactive chemicals, can be used as a neuroprotective pharmacological source for the development of effective medications to treat a range of diseases and disorders in the lives of people in the future.

## Figures and Tables

**Table 1:** LC-MS/MS mobile phase gradient elution condition.

No.	Time	Flow	A conc. (acetic acid in water)	B conc. (acetic acid in acetonitrile)
1		0.700	95.0	5.0
2	2	0.700	95.0	5.0
3	8	0.700	50.0	50.0
4	17	0.700	5.0	95.0
5	18	0.700	5.0	95.0
6	22	0.700	95.0	5.0
7	25	0.700	95.0	5.0

**Table 2:** Components identified in methanolic extract of *Costus spicatus* leaves (GC–MS method).

S. no	Rime	Area	Area (%)	A/H	Similarity	Compounds
1	7.338	119222	0.08	1.43	89	cis-2,6-Dimethyl-2,6-octadiene
2	7.583	123284	0.08	1.72	98	2-Pyrrolidinone
3	12.164	1695948	1.09	2.14	96	L-Proline, 5-oxo-, methyl ester
4	13.900	994721	0.64	4.42	96	beta-D-Glucopyranose, 1,6-anhydro-
5	14.043	865373	0.56	3.53	83	2,6,10-Dodecatrien-1-ol, 3,7,11-trimethyl-
6	14.207	2361135	1.52	7.03	67	Glutamine
7	14.291	4113957	2.65	9.98	92	Triacetylpseudotomatidine
8	14.546	670956	0.43	2.89	93	Dodecanoic acid
9	14.652	352118	0.23	2.87	88	3-Hydroxy-4-methoxybenzoic acid
10	15.768	667733	0.43	4.12	76	1H-Benzocyclohepten-9-ol, 2,4a-.beta.,5,6,7,8,9,9a-.beta.octahydro-3,5,5,9-.b
11	16.582	617146	0.4	2.97	95	(Dimethylamino)ethyl methacrylate
12	16.826	424570	0.27	2.4	94	Tetra-decanoic acid
13	17.082	371302	0.24	1.84	94	Loliolide
14	17.121	360213	0.23	1.53	94	1-Octadecene
15	17.587	516076	0.33	1.58	94	Neophytadiene
16	17.860	422712	0.27	3.4	82	Phthalic acid, 2-cyclohexylethyl isobutyl ester
17	17.976	301675	0.19	2.9	89	8-Octadecanone
18	18.390	1152119	0.74	2	90	trans-Geranylgeraniol
19	19.010	2182060	1.41	2.49	92	l-(+)-Ascorbic acid 2,6-dihexadecanoate
20	19.348	481160	0.31	2.24	86	Bromoacetic acid, octadecyl ester
21	21.011	499353	0.32	5.84	52	7-Methyl-8-oxo-1,2,3,4-tetrahydro-8H-pyrimido[1,2-a] pyrimidine
22	21.092	641616	0.41	3.19	97	Phytol
23	21.651	1833046	1.18	9.74	70	7-Methyl-6-oxo-1,2,3,4-tetrahydro-6H-pyrimido[1,2-a] pyrimidine
24	27.573	336459	0.22	4.48	91	1,6,10,14,18,22-Tetracosahexaen-3-ol, 2,6,10,15,19,23-hexamethyl-, (all-E)-
25	30.727	401517	0.26	3.35	81	Hexadecanoic acid, 2-hydroxy-1-(hydroxymethyl)ethyl ester
26	31.020	732765	0.47	8.29	70	Hexadecanoic acid, 1-(hydroxymethyl)-1,2-ethanediyl ester
27	31.240	2671224	1.72	7.85	79	Stigmasterol
28	31.494	549212	0.35	2.68	91	Bis (2-ethylhexyl) phthalate
29	32.553	768973	0.5	6.47	40	1-Coprosten-3-one semicarbazone
30	32.694	2511791	1.62	8.04	80	Campesterol
31	32.847	661525	0.43	5.01	35	Hexadecanoic acid, 2-[(1-oxododecyl) oxy]-1,3-propanediyl ester
32	33.097	2859778	1.84	9.38	51	Lanost-8-ene-7,11-dione
33	33.227	3685863	2.38	13.06	44	9, 19-Cyclolanostan-3-ol, acetate, (3.beta)-
34	33.387	979438	0.63	4.73	41	Androsta [17-16-b] furan-5′-imine, 4′-methylene-3-methoxy-N-cyclohexyl-
35	33.473	792096	0.51	3.04	52	Androsta [17-16-b] furan-5′-imine, 4′-methylene-3-methoxy-N-cyclohexyl-
36	33.689	116836	7.53	9.44	87	Stigmasterol
37	34.107	18996	12.25	19.1	79	gamma-Sitosterol
38	34.233	2738002	1.77	4.4	41	Triacontanoic acid
39	34.437	8445280	5.44	11.43	62	i-Propyl 9-octadecenoate (Z)
40	34.627	4999150	3.22	8.04	66	Methylprednisolone acetate
41	34.873	1754580	11.31	4.28	90	13-Docosenamide, (Z)-
42	34.967	3020987	1.95	5.91	41	Acetic acid, (4-chlorophenoxy)-, octadecyl ester
43	35.264	5761346	3.71	13.8	56	Triacetylpseudotomatidine
44	35.560	146836	9.47	11.34	85	gamma-Sitosterol
45	35.939	4935635	3.18	8.66	80	alpha-Tocospiro B
46	36.153	477538	0.31	7.06	42	(Z)-1,3-Dimethoxypropan-2-yl octadec-11-enoate
47	36.553	434125	0.28	4.19	68	9-Octadecenoic acid, 2-(octadecyloxy)ethyl ester
48	36.693	2339837	1.51	7.8	60	Ursa-12, 20 (30)-dien-28-oic acid, 2, 3, 23-trihydroxy (2. alpha. 3. beta., 4. alpha)
49	37.240	1001242	6.45	4.31	96	delta-Tocopherol
50	38.159	368046	0.24	5.93	49	3-Debenzoyloxy-anhydrocarpesteerol
51	38.736	2199655	1.42	4.48	89	beta-Tocopherol
52	39.054	7764968	5.02	5.1	94	gamma-Tocopherol
		1.55E + 08	100			

**Table 3:** Components identified in ethanolic extract of *Costus spicatus* leaves (GC–MS method).

S. no.	R. time	Area	Area (%)	A/H	Similarity	Compound
1	7.343	48378	0.01	1.08	88	cis-2,6-Dimethyl-2,6-octadiene
2	8.723	54660	0.01	1.83	91	Succinimide
3	11.354	29338	0.01	1.64	74	2-Methoxy-4-vinylphenol
4	13.004	200505	0.03	1.67	96	2-Pyrrolidinone, 5-(cyclohexylmethyl)-
5	13.068	114549	0.02	1.49	82	Geranyl vinyl ether
6	13.821	358542	0.06	3.15	93	beta-D-Glucopyranose, 1,6-anhydro-
7	14.047	197369	0.03	1.67	84	2,6,10-Dodecatrien-1-ol, 3,7,11-trimethyl-
8	14.571	934804	0.16	2.35	95	Dodecanoic acid
9	14.653	136506	0.02	2.59	65	1-Cyclohexene-1-ethanol, 2,6,6-trimethyl-
10	14.728	113578	0.02	2.05	83	Fumaric acid, ethyl 2-methylallyl ester
11	14.851	165907	0.03	4.14	68	7-Tetradecene
12	14.942	223171	0.04	1.8	95	Hexadecane
13	15.174	143018	0.02	2.04	80	5-Isopropenyl-2-methylcyclopent-1-enecarboxaldehyde
14	15.761	157134	0.03	2.11	80	2-Butanone, 4-(2,6,6-trimethyl-1-cyclohexen-1-yl)-
15	16.277	192904	0.03	2.8	84	2-Cyclohexen-1-one, 4-(3-hydroxybutyl)-3,5,5-trimethyl-
16	16.835	121014	0.02	2.55	85	Tetra-decanoic acid
17	17.003	142078	0.02	2.41	81	1,4-Naphthalenedione, 2,3,6-trimethyl-
18	17.083	624763	0.11	2.14	95	6-Hydroxy-4,4,7a-trimethyl-5,6,7,7a-tetrahydrobenzofuran-2(4H)-one
19	17.199	254246	0.04	1.84	93	Heptadecane, 2,6,10,15-tetramethyl-
20	17.593	103338	0.18	1.49	96	Neophytadiene
21	17.647	190180	0.03	1.55	92	2-Pentadecanone, 6,10,14-trimethyl-
22	17.851	565748	0.1	2.74	81	Cyclo-propanenonanoic acid, 2-[(2-butylcyclopropyl)methyl]-, methyl ester
23	18.044	596642	0.1	1.91	94	Neophytadiene
24	18.127	199106	0.03	3.53	75	2,6,10-Dodecatrien-1-ol, 3,7,11-trimethyl-
25	18.310	132915	0.02	3.6	76	Undec-10-ynoic acid, tridec-2-yn-1-yl ester
26	18.395	1832187	0.32	1.78	89	Hexadeca-2,6,10,14-tetraen-1-ol, 3,7,11,16-tetramethyl-
27	18.798	157401	0.03	2.36	93	1-Hexadecen-3-ol, 3,5,11,15-tetramethyl-
28	19.015	491234	0.08	3.14	90	l-(+)-Ascorbic acid 2,6-dihexadecanoate
29	19.113	234230	0.04	4.96	71	2,6,10,14,18-Pentamethyl-2,6,10,14,18-eicosapentaene
30	19.226	273526	0.05	2.48	86	Hexanoic acid, anhydride
31	19.316	793232	0.14	2.34	94	Hexadecenoic acid, ethyl ester
32	19.361	235988	0.04	1.84	85	trans-Geranylgeraniol
33	19.426	203360	0.04	3.05	88	Eicosane
34	21.103	781398	0.13	3.02	94	Phytol
35	21.943	144102	0.02	2.93	90	trans, trans-9,12-Octadecadienoic acid, propyl ester
36	22.072	231774	0.04	2.98	81	2-Octylcyclopropene-1-heptanol
37	26.798	248364	0.04	4.26	93	4,8,12,16-Tetramethylheptadecan-4-olide
38	27.593	731311	0.13	5.41	93	Hexadeca-2,6,10,14-tetraen-1-ol, 3,7,11,16-tetramethyl-
39	30.040	350096	0.06	4.93	78	2,6,10,14,18-Pentamethyl-2,6,10,14,18-eicosapentaene
40	30.737	632694	0.11	3.76	80	Hexadecanoic acid, 2-hydroxy-1-(hydroxymethyl)ethyl ester
41	31.013	153407	0.03	4.04	64	Glycerol 1-palmitate
42	31.227	233209	0.04	3.94	76	Hexadecanoic acid, 2-hydroxy-1-(hydroxymethyl)ethyl ester
43	31.508	455842	0.08	2.83	90	Bis (2-ethylhexyl) phthalate
44	31.707	712591	0.12	7.77	40	1-Phenanthrenecarboxylic acid, 1,2,3,4,4a,4b,5,9,10,10a-decahydro-1,4a-dimethyl-7-(1-methylethyl)-, ethyl ester, [1R-(45 32.094 6946637 1.20 21.65 63 hexadecanoic acid, 2-[(1-oxododecyl)oxy]-1,3-propanediyl ester
46	33.280	439712	7.58	40.74	68	Octadecanoic acid, 3-[(1-oxohexadecyl)oxy]-2-[(1-oxotetradecyl)oxy]propyl ester
47	33.583	256440	4.42	15.44	72	Octadecanoic acid, 3-[(1-oxohexadecyl)oxy]-2-[(1-oxotetradecyl)oxy]propyl ester
48	34.039	414914	7.15	21.07	59	9-Octadecenoic acid (Z)-, 3-[(1-oxohexadecyl)oxy]-2-[(1-oxooctadecyl)oxy]propyl ester
49	34.160	382995	6.6	20.72	49	9-Octadecenoic acid (Z)-, 3-[(1-oxohexadecyl)oxy]-2-[(1-oxooctadecyl)oxy]propyl ester
50	34.880	392753	6.77	19.79	85	13-Docosenamide, (Z)-
51	35.040	168510	2.9	10.65	44	Hexadecanoic acid, 2-[(1-oxododecyl)oxy]-1,3-propanediyl ester
52	35.661	777982	13.41	37	70	alpha-Tocospiro B
53	35.965	370832	6.39	17.75	67	alpha-Tocospiro A
54	36.580	427023	7.36	22.77	62	9-Octadecenoic acid (Z)-, 3-[(1-oxohexadecyl)oxy]-2-[(1-oxooctadecyl)oxy]propyl ester
55	36.797	281678	4.85	16.7	58	Hexadecanoic acid, 2-[(1-oxododecyl)oxy]-1,3-propanediyl ester
56	37.259	475530	8.2	11.29	97	delta-Tocopherol
57	37.760	356769	6.15	22.1	40	Fumaric acid, decyl 2-fluorophenyl ester
58	37.913	187253	3.23	11.76	36	Glycine, N-(cyclobutylcarbonyl)-, hexadecyl ester
59	38.049	275855	4.75	17.67	40	Glycine, N-(cyclobutylcarbonyl)-, heptadecyl ester
60	38.540	147917	2.55	16.96	33	9-Octadecenoic acid (Z)-, 3-[(1-oxohexadecyl)oxy]-2-[(1oxooctadecyl)oxy]propyl ester
61	38.758	121119	2.09	9.42	93	beta-Tocopherol
62	39.075	914507	1.58	5.69	95	gamma-Tocopherol
63	39.420	577783	0.1	10.18	45	9-Octadecenoic acid (Z)-, 2-(octadecyloxy) ethy

**Table 4:** Antioxidant properties of *Costus spicatus* using different solvents examined under different parameters.

Parameter	Ethanol	Methanol	Alcoholic aquas	Aquas
DPPH assay (mg/mL)	1.02 ± 0.02	1.25 ± 0.02	2.05 ± 0.02	2.24 ± 0.02
Hydroxyl radical scavenging activity (mg/mL)	0.84 ± 0.002	0.75 ± 0.002	1.98 ± 0.002	1.06 ± 0.002
Lipid peroxidation (%)	36.02 ± 5	30.27 ± 5	28.81 ± 5	42.84 ± 5

**Table 5:** LC-MS/MS analysis of aqueous leaf extract of *C. spicatus* at positive ESI.

No.	RT	(+) ESI-MS m/z/ (+) ESI-MS/MS m/z	Compound name	Chemical classification
1.	4.26	359.0658 [M + Na]^+^	Caffeoyl shikimate (tentatively identified)	Phenols
2.	5.15	611.1602 [M + H]^+^	Rutin (tentatively identified)	Flavonoids
3.	6.32	901.6133	Protodeltonin (tentatively identified)	Steroid saponin
4.	6.77	901.4246	Methylprotogracillin (tentatively identified)	Flavonoids
5.	7.21	415.2805	Pseudoprotodioscin (tentatively identified)	Steroid saponin

**Table 6:** Docking interactions of compound and alpha-synuclein active site residues.

S. no.	Ligand ID	Pose	Score	RMSD LB	RMSD UB
1	Ligand29	1	−7.4	0	0
2	Ligand27	1	−7.2	0	0
3	Ligand37	1	−6.8	0	0
4	Ligand40	1	−6.8	0	0
5	Ligand47	1	−6.7	0	0
6	Ligand32	1	−6.6	0	0
7	Ligand493D	1	−6.5	0	0
8	Ligand16	1	−6.4	0	0
9	Ligand18	1	−6.3	0	0
10	Ligand48	1	−6.3	0	0
11	Ligand10	1	−6.2	0	0
12	Ligand3	1	−6.1	0	0
13	Ligand4	1	−6.1	0	0
14	Ligand20	1	−6	0	0
15	Ligand23	1	−5.6	0	0
16	Ligand21	1	−5.5	0	0
17	Ligand45	1	−5.5	0	0
18	Ligand5	1	−5.5	0	0
19	Ligand9	1	−5.4	0	0
20	Ligand513D	1	−5.3	0	0
21	Ligand13	1	−5.2	0	0
22	Ligand6	1	−5.1	0	0
23	Ligand17	1	−5	0	0
24	Ligand15	1	−4.7	0	0
25	Ligand11	1	−4.6	0	0
26	Ligand14	1	−4.5	0	0
27	Ligand22	1	−4.5	0	0
28	Ligand7	1	−4.5	0	0
29	Ligand1	1	−4.4	0	0
30	Ligand8	1	−4.4	0	0
31	Ligand12	1	−4.3	0	0
32	Ligand2	1	−3.6	0	0

**Table 7:** Docking result between the selected compound and alpha-synuclein with their interactions.

S. no.	Ligand ID	Score	Compound name
1	Ligand29	−7.4	Androsta [17-16-b]furan-5′-imine, 4′-methylene-3-methoxy-N-cyclohexyl-
2	Ligand27	−7.2	9, 19-Cyclolanostan-3-ol, acetate, (3.beta.)-
3	Ligand37	−6.8	i-Propyl 9-octadecenoate (Z)
4	Ligand40	−6.8	l-(+)-Ascorbic acid 2,6-dihexadecanoate (2d)

## Data Availability

The datasets used and/or analysed during the current study are available from the corresponding author on reasonable request.
